# Clinical and laboratory risk factors for pulmonary tuberculosis recurrence in three pooled Indian cohorts

**DOI:** 10.3389/ftubr.2024.1433975

**Published:** 2024-07-12

**Authors:** Sonya Krishnan, Nikhil Gupte, Mandar Paradkar, Akshay Gupte, Mrunmayi Naik, Swapnil Raskar, Nishi Suryavanshi, Neeta Pradhan, Sanjay Gaikwad, Rajesh Karyakarte, Rahul Lokhande, Elizabeth Hanna Luke, Kannan Thiruvengadam, Chandrasekaran Padmapriyadarsini, Tushar Sahasrabudhe, Madhusudan Barthwal, ArjunLal Kakrani, Vijay Viswanathan, Hardy Kornfeld, Amita Gupta, Jonathan E. Golub, Vidya Mave

**Affiliations:** ^1^Department of Medicine, Johns Hopkins University School of Medicine, Baltimore, MD, United States; ^2^Johns Hopkins Center for Infectious Diseases in India, Pune, India; ^3^BJ Government Medical College, Johns Hopkins Clinical Research Site, Pune, India; ^4^Department of Global Health, Boston University School of Public Health, Boston, MA, United States; ^5^BJ Government Medical College and Sassoon General Hospitals, Pune, India; ^6^Indian Council of Medical Research, National Institute for Research in Tuberculosis, Chennai, India; ^7^Dr. D. Y. Patil Medical College, Hospital and Research Centre, Pune, India; ^8^M. V. Hospital for Diabetes, Chennai, India; ^9^Department of Medicine, University of Massachusetts Chan Medical School, Worcester, MA, United States; ^10^Center for Tuberculosis Research, School of Medicine, Johns Hopkins University, Baltimore, MD, United States

**Keywords:** tuberculosis, recurrence, risk factors, adverse outcome, epidemiology (EPI)

## Abstract

Some individuals with drug-susceptible pulmonary tuberculosis (PTB) experience tuberculosis recurrence. To evaluate the incidence of and risk factors for recurrence following completion of antituberculosis therapy, we pooled data from three prospective observational Indian PTB cohorts with 1,164 individuals ≥14 years old included in our analysis. Ninety-five (8%) experienced recurrence, with an 8.5 cases/100 person-years recurrence incidence rate (95% confidence interval 6.9–10.3) and a median time to recurrence of 6 months. From multivariable logistic regression, month 2 culture positivity (aHR 2.06; 95% CI 1.17–3.63), body mass index (BMI) < 17 mg/kg^2^ (aHR 1.7; 95% CI 1.1–2.8), and male sex (aHR 1.92; 95% CI 1.05–3.51) were independent recurrence risk factors. Understanding risk factors for TB recurrence could enable clinicians to identify patients at risk for recurrence during antituberculosis therapy and may be used to alter patient care strategies, such as more frequent monitoring post-treatment for high-risk individuals.

## Introduction

Of the 7.5 million people diagnosed with tuberculosis (TB) globally in 2022, India had the highest proportion of TB, accounting for more than 30% of cases (1). Despite a ≥90% End TB Strategy goal treatment success rate (1, 2), programmatic settings often achieve suboptimal outcomes, including recurrence, which occurs after individuals have successfully completed antituberculosis therapy (ATT) and are deemed cured (3). The advent of genotyping of *Mycobacterium tuberculosis* (*Mtb*) has allowed for further classification of recurrence into either relapse of disease or reinfection (4), with reinfection more likely in hyperendemic areas (5, 6). Risk factors associated with TB recurrence include higher smear grade at baseline and month 2 of therapy (7, 8), HIV status (9), alcohol and tobacco use (10), month 2 culture positivity, and baseline chest xX-ray (CXR) cavitation (11, 12).

While studies have primarily focused on baseline risk factors, few have incorporated microbiological data throughout ATT and have not evaluated the timing of recurrence post-ATT completion. Identifying independent TB recurrence risk factors could enable clinicians to accurately identify these patients and augment treatment or post-treatment monitoring strategies (13). Using data from three Indian drug-sensitive pulmonary TB (PTB) observational cohorts, we evaluated recurrence incidence rates and risk factors, using data collected throughout ATT.

## Materials and methods

### Study design

We leveraged data from three Indian observational cohorts designed to assess the incidence rate of and risk factors for unfavorable TB treatment outcomes: Cohort for Tuberculosis Research by the Indo-US Medical Partnership (C-TRIUMPh) (14), The Effects of Diabetes on Tuberculosis Severity (EDOTS) (15), and The Impact of Diabetes on TB Treatment Outcomes (TB-DM) (16). These cohorts enrolled microbiologically confirmed or clinically diagnosed PTB; participants initiated standard of care ATT, consisting of 2 months isoniazid, rifampin, pyrazinamide, and ethambutol either thrice weekly or daily followed by 4 months of isoniazid and rifampin, according to India's National Tuberculosis Elimination Program (NTEP) guidelines (17). Before 2017, ATT was administered as thrice-weekly directly observed therapy; After 2017, NTEP guidelines shifted to daily self-administered regimens.

### Study population

Participants were recruited from local tuberculosis units to four clinical research sites in Pune or Chennai. Those with newly diagnosed rifampin-susceptible PTB aged ≥ 14 years were included in this study. They had signs or symptoms consistent with TB disease: cough, hemoptysis, fever, weight loss, fatigue, night sweats, or pleuritic chest pain. They also had CXR findings consistent with TB, and/or were sputum smear positive by microscopy or by rapid diagnostic test (i.e., GeneXpert). Participants who had received 1 week (daily or intermittent doses) of any drugs with anti-TB activity within 30 days were excluded. TB-DM and EDOTS excluded people with HIV. Supplementary material include detailed information on enrollment dates, study sites, and cohort-specific inclusion and exclusion. All participants were followed during treatment and for up to 12 months following treatment completion. Standardized clinical assessments, demographics, sputum sampling, and outcome assessments were collected at baseline and months 2, 6, 12, and 18.

### Primary outcomes

TB cure was defined as ≥2 consecutive respiratory sputum cultures negative for *Mtb* following the completion of 6 months of ATT with continued resolution of symptoms and negative mycobacteriology assessments (smear and culture). Following successful treatment completion, TB recurrence was defined as microbiological recurrence with *Mtb* growth on culture [either liquid Mycobacterium Growth Incubator Tube (MGIT) or solid Löwenstein-Jensen (LJ) culture media], or clinical recurrence based on symptoms suggestive of TB and acid-fast bacilli on smear microscopy. TB recurrence included cases of both reinfection and relapse, as whole genome sequencing of isolates to compare to baseline samples was not available.

### Variable definitions

Smoking was defined as a history of ever smoking tobacco products (including current and former smokers), and alcohol use was defined as any reported history of alcohol use. Diabetes was defined as hemoglobin A1c > 6.5%, a random blood glucose > 200 mg/dL, or a prior diagnosis of diabetes on antidiabetic medication. A Timika CXR score was calculated as a summation of the percentage of lungs involved from 0 to 100% and an additional 40 points added for the presence of cavity (18).

### Primary statistical analyses

The recurrence incidence rate was calculated as the number of individuals with recurrence divided by total person-time at risk per 100 person-years [Poisson exact 95% confidence intervals (CI)]. Variables associated with recurrence were evaluated using univariable Cox Proportional Hazards regression. To account for confounders identified a priori based on a literature review, we used multivariable Cox Proportional Hazards regression and visualized results using Forest plots. Differences between categorial variables (summarized as proportions) were assessed (Fisher's exact test). Continuous variables were summarized as medians with interquartile ranges (IQR) or means with standard deviation and were compared (Wilcoxon rank-sum test or *t*-test). The level of significance was assessed at 5%. Statistical analyses were conducted using Stata Version 17 (StataCorp, College Station, TX, USA) and RStudio Version 4.2.2 (Rstudio, Boston, MA, USA). To generate a recurrence prediction model, a priori we selected seven candidate predictors: age, alcohol use, sex, smoking history, month 2 culture positivity, month 2 smear positivity, and body mass index (BMI), based on prior TB prediction models (19) and co-author clinical input. Data was split into a training model (60%) and a validation model (40%). Model discrimination was evaluated by receiver operating characteristic (ROC) curve, area under the curve (AUC), and c-statistic.

### Ethics statement

Ethics Committee/Institutional Review Board approval was obtained from each Indian participating site, Johns Hopkins University and the University of Massachusetts. Informed consent was obtained from all study participants.

## Results

Of the 2,062 individuals enrolled in TB-DM, C-TRIUMPh, and EDOTS, 1,516 completed treatment and achieved cure; 1,164 individuals ≥14 years old were included in our analysis (Supplementary Figure 1), contributing a cumulative 1,118 person-years of follow-up. Individuals who experienced treatment failure, death, had baseline rifampin resistance, were lost to follow-up prior to 6 months, or withdrew, were excluded. Ninety-five (8%) experienced recurrence. Individuals who experienced recurrence were more likely males, from Pune, with a lower BMI, an alcohol use history, and had a positive month 2 smear or culture result (Table 1). The overall recurrence incidence rate was 8.5/100 person-years (95% CI 6.9–10.3), which occurred 179 days (median, IQR: 115–271) after ATT completion. Recurrence occurred in the following intervals after ATT completion: *n* = 18 at ≤ 3 months, *n* = 30 at >3– ≤ 6 months, *n* = 23 at >6– ≤ 9 months, and *n* = 24 at ≥9 months. Eighteen individuals experienced clinical recurrence, whereas 57 individuals experienced microbiologically confirmed recurrence (Supplementary Table 1).

**Table 1:** Characteristics of the study population throughout antituberculosis therapy and incidence rate of recurrence by key characteristics.

**Characteristic**	**Categories**	**Overall proportion**	**Recurrence^a^ (*n* = 95; *n*, %)**	**Recurrence incidence rate/100 person-years (95% CI)**	***P*-value^b^**
**Baseline characteristics**					
Study city	Chennai	506 (43%)	31 (33%)	5.3 (3.7–7.7)	0.03
	Pune	658 (57%)	64 (67%)	11.8 (9.1–15.0)	
Sex	Male	781 (67%)	75 (79%)	10.0 (7.9–12.6)	0.01
	Female	383 (33%)	20 (21%)	5.4 (3.3–8.3)	
Age (years)	14– < 25	264 (23%)	24 (25%)	9.6 (6.2–14.3)	0.004
	25– < 45	509 (44%)	53 (56%)	11.1 (8.3–14.5)	
	≥45	391 (33%)	18 (19%)	4.6 (2.7–7.3)	
Education (years)	Illiterate	130 (11%)	12 (13%)	8.9 (4.6–15.6)	0.46
	0– < 5	528 (45%)	19 (20%)	11.0 (6.6–17.2)	
	5– < 10	176 (15%)	41 (43%)	8.1 (5.8–10.9)	
	≥ 10	328 (28%)	23 (24%)	7.7 (4.9–11.5)	
Dwelling condition^c^	Rural	176 (15%)	11 (12%)	5.4 (2.7–9.7)
	Urban/Periurban	623 (54%)	64 (67%)	10.2 (7.8–13.0)	0.23
Body mass index (kg/m^2^)	17– < 25	667 (57%)	42 (44%)	6.5 (4.7–8.8)	
	< 17	414 (36%)	50 (53%)	12.8 (9.5–16.9)	
	≥25	81 (7%)	3 (3%)	3.6 (0.8–10.7)	0.001
Smoking history	Never	799 (69%)	63 (66%)	8.4 (6.5–10.8)	0.65
	Ever	291 (25%)	25 (26%)	9.2 (5.9–13.5)	
Alcohol use	None	684 (59%)	45 (47%)	7.1 (5.2–9.5)	0.04
	Any	439 (38%)	44 (46%)	10.1 (7.3–13.5)	
Diabetes	No	695 (60%)	65 (68%)	9.7 (7.5–12.3)	0.08
	Yes	469 (40%)	30 (32%)	6.7 (4.5–9.6)	
Smear	Negative	259 (22%)	14 (15%)	5.5 (3.0–9.2)	0.07
	Positive	903 (78%)	81 (85%)	9.4 (7.5–11.7)	
Culture	Negative	117 (10%)	5 (5%)	4.1 (1.3–9.5)	0.11
	Positive	1,041 (89%)	90 (95%)	9.1 (7.3–11.2)	
Chest X-ray cavity	Absent	571 (49%)	43 (45%)	7.2 (4.6–10.7)	> 0.95
	Present	321 (28%)	24 (25%)	7.6 (5.5–10.2)	
Smear grade	1+	217 (19%)	16 (17%)	7.2 (4.1–11.7)	0.24
	2+	199 (17%)	20 (21%)	9.9 (6.0–15.2)	
	3+	107 (9%)	13 (14%)	14.7 (7.8–25.1)	
	Negative	268 (23%)	17 (18%)	6.6 (3.8–10.6)	
	Scanty	70 (6%)	9 (9%)	15.4 (7.0–29.2)	
Antituberculosis treatment regimen	Daily	181 (16%)	11 (12%)	8.2 (4.1–14.6)	0.37
	Thrice weekly	976 (84%)	82 (86%)	8.4 (6.7–10.4)	
**Post antituberculosis treatment initiation characteristics**					
Month 2 smear	Negative	881 (76%)	57 (60%)	6.6 (5.0–8.6)	0.006
	Positive	110 (9%)	16 (17%)	13.6 (7.8–22.1)	
Month 2 culture	Negative	829 (71%)	50 (53%)	6.2 (4.6–8.2)	0.001
	Positive	119 (10%)	18 (19%)	14.9 (8.8–23.5)	
Month 2 smear grade	1+	41 (4%)	4 (4%)	7.6 (2.1–19.4)	0.37
	2+	2 (0%)	0 (–)	–	
	3+	0 (–)	0 (–)	–	
	Negative	725 (62%)	55 (58%)	7.9 (5.9–10.3)	
	Scanty	12 (1%)	2 (2%)	24.2 (2.9–87.4)	
Month 6 chest X-ray cavity	Absent	412 (35%)	23 (24%)	14.6 (7.0–26.8)	0.02
	Present	73 (6%)	10 (11%)	5.9 (3.7–8.9)	

^a^Recurrence of tuberculosis defined as *Mycobacterium tuberculosis* growth on culture, or symptoms suggestive of TB and AFB detected on smear microscopy, following successful treatment completion.

^b^*P*-values assessed using Fisher's exact test.

^c^Dwelling condition data not available for The Effects of Diabetes on Tuberculosis Severity (EDOTS) cohort.

**Figure 1:**
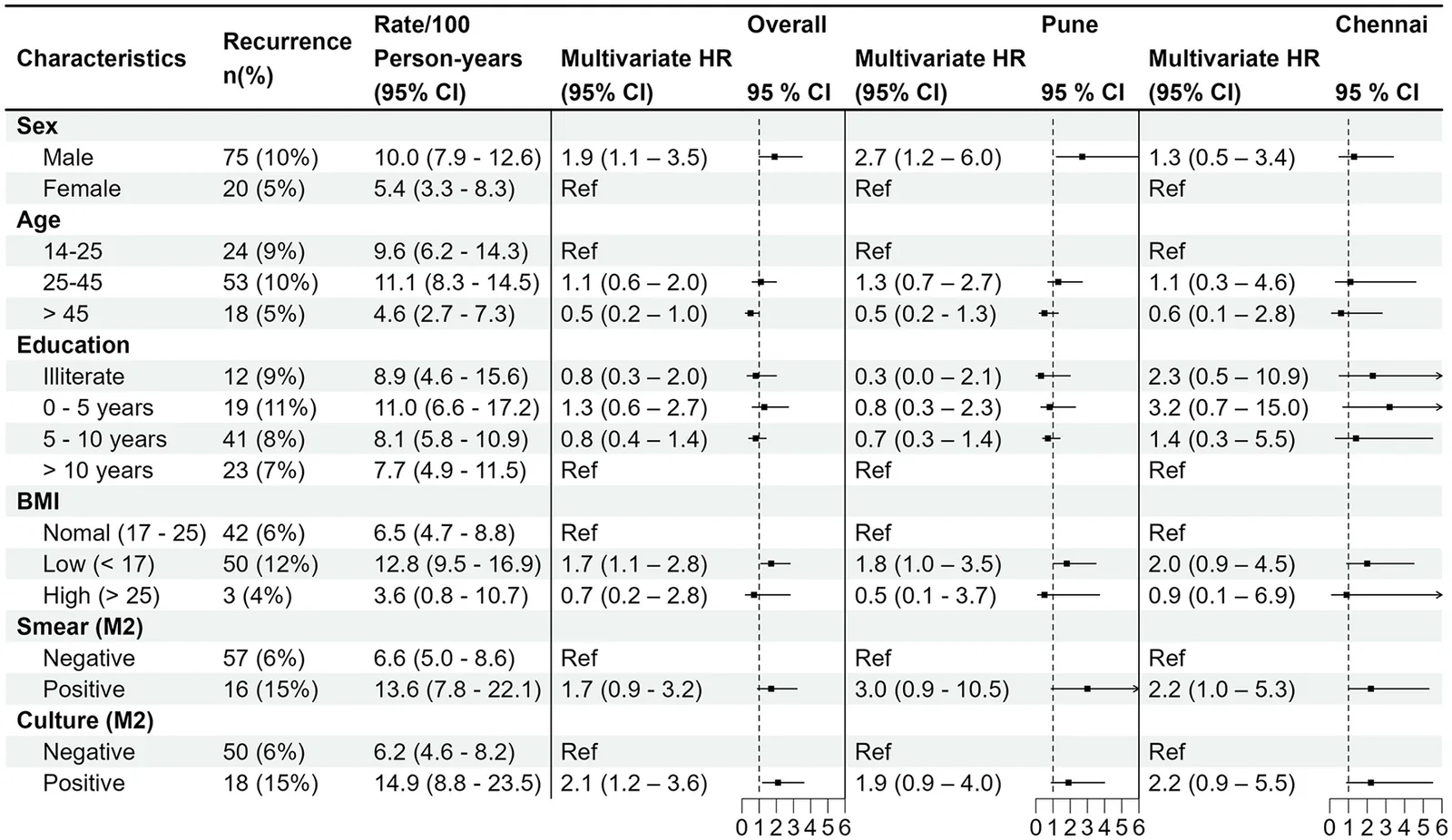
Forest plot showing the overall multivariable hazards of recurrence, adjusting for smoking history, alcohol use history, baseline smear result, baseline culture, baseline cavity, baseline X-ray score, antituberculosis therapy dosing regimen (thrice weekly vs. daily). City-specific stratification (Pune vs. Chennai) also shown. HR, hazard ratio; BMI, body mass index (kg/m^2^); M2, month 2.

Univariable regression analysis showed male sex, age > 45 years, BMI < 17 kg/m^2^, positive month 2 smear, positive month 2 culture, and month 6 cavity on CXR were significantly associated with increased TB recurrence hazards (Supplementary Table 2). Multivariable regression analysis identified male sex (aHR 1.92; 95% CI 1.05–3.51), BMI < 17 kg/m^2^ (aHR 1.72; 95% CI 1.1–2.8), and month 2 culture positivity (aHR 2.06; 95% CI 1.17–3.63) as independent risk factors for recurrence (Supplementary Table 2; Figure 1). When stratified by site, no city-specific associations emerged (Figure 1). A stratified analysis comparing males with and without recurrence revealed no differences in modifiable habits such as alcohol use or smoking history (Supplementary Table 3).

We attempted to create a recurrence prediction model with age, alcohol use, sex, smoking history, month 2 culture positivity, month 2 smear positivity, and BMI as predictors, but the model performed poorly with an AUC of 0.66 (sensitivity 85%; specificity 37%).

## Discussion

This analysis of Indian adolescents and adults with drug-sensitive pulmonary tuberculosis, enrolled in three prospective observational Indian cohorts, provides key insights into the time to recurrence following treatment completion, as many previously published studies on recurrence do not include this information. This study also highlights differences among individuals who achieved a sustained cure following treatment completion compared to those who experienced recurrence. We observed that a high proportion (8%) experienced TB recurrence with an overall incidence rate of 8.5 cases/100 person-years (95% CI 6.9–10.3) and a 6-month median time to recurrence following treatment completion. Recurrence events occurred throughout ATT completion follow-up, without a 3-month timeframe containing the majority of events. Male sex, BMI < 17 kg/m^2^, and month 2 culture positivity were key independent recurrence risk factors. The association of male sex remained after accounting for confounding behavioral factors associated with increased risk of adverse TB treatment outcomes, such as smoking and alcohol use, habits which were far more common in men in this cohort (10, 20, 21). While the increased risk of tuberculosis disease in males is well-documented (22), these findings highlight the increased susceptibility of the male sex in a specific adverse TB treatment outcome: recurrence. Consistent with prior studies, month 2 culture positivity was also an important recurrence risk factor (23). With a low BMI cutoff of < 17 kg/m^2^, BMI was associated with recurrence on multivariable regression analysis. Undernutrition is a well-established risk factor for the development of TB disease (13, 24). There have been several studies suggesting that Asian Indians have more body fat relative to weight than their Caucasian counterparts. Thus, the conventional low BMI cut-off of < 18.5 kg/m^2^, derived from mortality statistics from primarily white populations, may poorly predict risk of disease in other groups (25–27). For diabetes, for example, a lower BMI cut-off point is recommended for screening for Asians by the American Diabetes Association (28). Our study suggests that use of a lower BMI cut-off when assessing for recurrence risk could be important in an Asian Indian population. We demonstrated some challenges with creating a recurrence prediction model despite a robust number of events. Given that recurrence occurred a median of 6 months after cure, it could be that traditional baseline epidemiologic risk factors are not always accurate predictors of recurrence, and more data at ATT completion is needed for improved prediction.

Our study has some limitations. We could not evaluate whether recurrence represented reinfection or relapse as genotyping comparing the *Mtb* strains at baseline and at the time of recurrence was not available (29). At the time the studies were started, thrice weekly ATT was the national program standard, and this shifted to daily ATT while the studies were being conducted. Although 85% received thrice weekly, rather than daily ATT, per the standard of care at the time of the study, we continued to observe increased recurrence hazards after adjusting for this variable; this could have contributed to our higher recurrence rates. While recurrence is known to occur more commonly in people with HIV, we were unable to assess this risk factor due to a low HIV prevalence (and exclusion by two cohorts). Finally, while we are unable to broadly extrapolate our findings to populations with recurrence of pulmonary TB outside of Pune and Chennai, India, given the nature of the cohorts, many of the risk factors found in our study are in line with previously published risk factors; a systematic meta-analysis including more heterogenous populations may yield different results.

In conclusion, we describe a high proportion of recurrence in our Indian cohort of individuals treated for drug-sensitive PTB. Interestingly, recurrence occurred throughout 9 months of post-ATT follow-up, suggesting that there is not a specific timeframe where monitoring for recurrence would be most useful. Given that India accounts for the highest burden of TB cases globally, a comprehensive understanding of risk factors for recurrence in Indian patients is critical, and our analysis could provide insights to clinicians to identify patients at risk of recurrence during ATT and may be used to alter patient care strategies, such as prolongation of ATT or more frequent monitoring post treatment for high-risk individuals, which could include clinical assessments, sputum culture, or imaging.

## Data availability statement

The data analyzed in this study is subject to the following licenses/restrictions: Identifiable information. De-identified dataset available upon request. Requests to access these datasets should be directed to https://reportindia.org.

## Ethics statement

The studies involving humans were approved by Johns Hopkins University, University of Massachusetts, Dr. D. Y. Patil Medical College (DYPMC), Byramjee-Jeejeebhoy Government Medical College-Sassoon General Hospitals (BJGMC-SGH), National Institute for Research in Tuberculosis, and Prof. M. V. Diabetes Research Center (MVDRC). The studies were conducted in accordance with the local legislation and institutional requirements. Written informed consent for participation in this study was provided by the participants' legal guardians/next of kin.

## Author contributions

SK: Conceptualization, Data curation, Formal analysis, Investigation, Methodology, Supervision, Writing – original draft, Writing – review & editing. NG: Conceptualization, Data curation, Formal analysis, Investigation, Methodology, Project administration, Validation, Visualization, Writing – review & editing. MP: Writing – review & editing, Data curation, Project administration. AGupte: Writing – review & editing, Methodology. MN: Writing – review & editing, Formal analysis. SR: Writing – review & editing, Data curation. NS: Writing – review & editing, Investigation. NP: Writing – review & editing, Investigation. SG: Writing – review & editing, Investigation. RK: Writing – review & editing, Investigation. RL: Writing – review & editing, Investigation. EH: Writing – review & editing, Investigation. KT: Writing – review & editing, Investigation. CP: Writing – review & editing, Investigation. TS: Writing – review & editing, Investigation. MB: Writing – review & editing, Investigation. AK: Writing – review & editing, Investigation. VV: Writing – review & editing, Investigation. HK: Writing – review & editing, Funding acquisition, Investigation. AGupta: Writing – review & editing, Conceptualization, Formal analysis, Funding acquisition, Investigation. JG: Investigation, Writing – review & editing, Funding acquisition. VM: Conceptualization, Funding acquisition, Investigation, Writing – review & editing.
