# Supplementary material for: Clinical and laboratory risk factors for pulmonary tuberculosis recurrence in three pooled Indian cohorts
Source: Front Tuberc. 2024 Jul 12;2:1433975. doi: 10.3389/ftubr.2024.1433975 (PMC12040415; doi:10.3389/ftubr.2024.1433975)
Supplement: Supplementary file 1 [file Data_Sheet_1.docx]

**SUPPLEMENTARY METHODS**

**Enrolment information**

The Impact of Diabetes on TB Treatment Outcomes (TB-DM), enrolled participants from December 2013-May 2019 at the Byramjee-Jeejeebhoy Government Medical College-Sassoon General Hospitals (BJGMC-SGH) clinical research site with Dr. D.Y. Patil Medical College (DYPMC) joining the study in 2016[1], both located in Pune, India. The second cohort, Cohort for TB Research by the Indo-US Medical Partnership Multicentric Prospective Observational Study (C-TRIUMPh), was conducted at BJGMC-SGH and National Institute for Research in Tuberculosis (NIRT) in Chennai, India[2] and enrolled participants from August 2014 – November 2018. The EDOTS cohort enrolled participants from January 2014 to July 2015 at the Prof.M.V. Diabetes Research Centre (MVDRC) located in Chennai.[3]

**Detailed Inclusion/Exclusion Criteria for Each Cohort**

EDOTS

Participants aged 25-60 were eligible if they had acid-fast bacilli and smear-positive results with a suspicion for TB and presented to 10 treatment units. They had to provide informed consent. They were excluded if they had prior incidence TB disease, had received over 7 days of antituberculosis therapy for the current TB episode, were pregnant or nursing, were HIV seropositive, had received immunosuppressive therapy, or more than 7 doses of a fluoroquinolone in the past 30 days.

C-TRIUMPH

Subject inclusion criteria included adults and children (<14 years of age) with newly diagnosed pulmonary TB or extrapulmonary TB presenting to 3 local treatment units, and met National TB Elimination Program Category-1 treatment criteria. They had to provide informed consent or assent, allow home visits, and live within a 50 kilometre radius of the treatment units. For pulmonary TB, they had to either be smear or Xpert positive at entry, or if they were smear negative at entry, they needed to be culture confirmed by 12 weeks. Subjects were excluded if they had critical illness with life expectancy of <1 month, weight of less than 5 kilograms, had a medical condition that could interfere with participation or interpretation of results, had received more that 1 week (5 daily or 3 intermittent doses) of multi-drug TB therapy in the preceding 30 days, or received more than 7 days of fluoroquinolone therapy or other drugs with anti-TB activity for any reason in the preceding 30 days.

TB-DM

Inclusion criteria included men and women 18 years and older with microbiologic confirmation of TB (either sputum specimen positive for acid-fast bacilli or GeneXpert, or culture positive who were anticipated to complete TB treatment at one of the study sites. They were defined as having diabetes mellitus based on one of the following: HbA1c ≥6.5%, Fasting Blood Glucose level (FG) ≥126 mg/dl, Random Blood Glucose Level ≥ 200 mg/dl, Diabetes diagnosis by self-report, or on diabetes medication. Exclusion criteria included infection with rifampin-resistant TB, HIV infection, and pregnancy.

**SUPPLEMENTARY RESULTS**

Abbreviations: Cohort for Tuberculosis Research by the Indo-US Medical Partnership, c-TRIUMPh; The Effects of Diabetes on Tuberculosis Severity, EDOTS; The Impact of Diabetes on TB Treatment Outcomes, TB-DM; Tuberculosis, TB; Pulmonary tuberculosis, PTB; Extrapulmonary tuberculosis, EPTB

**Supplemental Figure 1**. Flow diagram demonstrating the selection of individuals eligible for inclusion in this study.

**Supplementary Table 1.** Symptoms, imaging, and microbiological data for clinical recurrence versus microbiologically confirmed recurrence at the time of recurrence

|  | All Recurrence  N = 95 | Clinical Recurrence  N = 18 | Microbiologically confirmed Recurrence  N = 57 |
| --- | --- | --- | --- |
| Any Symptom | Yes : 38 (40%)  No : 25 (26%)  Not Available : 32 (34%) | Yes : 8 (44%)  No : 4 (22%)  Not Available : 6 (33%) | Yes : 30 (53%)  No : 21 (37%)  Not Available : 6 (11%) |
| Cough | Yes : 36 (38%)  No : 26 (27%)  Not Available : 33 (35%) | Yes : 8 (44%)  No : 4 (22%)  Not Available : 6 (33%) | Yes : 28 (49%)  No : 22 (39%)  Not Available : 7 (12%) |
| Fever | Yes : 23 (24%)  No : 40 (42%)  Not Available : 32 (34%) | Yes : 3 (17%)  No : 9 (50%)  Not Available : 6 (33%) | Yes : 20 (35%)  No : 31 (54%)  Not Available : 6 (11%) |
| Night Sweats | Yes : 10 (11%)  No : 53 (56%)  Not Available : 32 (34%) | Yes : 2 (11%)  No : 10 (56%)  Not Available : 6 (33%) | Yes : 8 (14%)  No : 43 (75%)  Not Available : 6 (11%) |
| Weight Loss | Yes : 18 (19%)  No : 44 (46%)  Not Available : 33 (35%) | Yes : 3 (17%)  No : 9 (50%)  Not Available : 6 (33%) | Yes : 15 (26%)  No : 35 (61%)  Not Available : 7 (12%) |
| Chest Xray Involvement  (Cavitation at 6 Month) | Yes : 5 (5%)  No : 32 (34%)  Not Available : 61 (65%) | Yes : 1 (6%)  No : 1 (6%)  Not Available : 16 (89%) | Yes : 4 (7%)  No : 11 (19%)  Not Available : 42 (74%) |
| AFB Smear Positive | Positive : 18 (19%)  Negative : 39 (41%)  Not Available : 38 (40%) | Positive : 0 (0%)  Negative : 12 (67%)  Not Available : 6 (33%) | Positive : 18 (32%)  Negative : 27 (47%)  Not Available : 12 (21%) |
| Culture Positive | Positive : 34 (36%)  Negative : 23 (24%)  Not Available : 38 (40%) | Positive : 0 (0%)  Negative : 12 (67%)  Not Available : 6 (33%) | Positive : 34 (60%)  Negative : 11 (19%)  Not Available : 12 (21%) |

**Supplementary Table 2.** Univariable and multivariable hazards of recurrence.

| Variables | Categories | Univariable | | Multivariable |  |
| --- | --- | --- | --- | --- | --- |
|  |  | **HR (p-value)** | **95% CI** | **HR (p-value)** | **95% CI** |
| Sex | Male  Female | 1.85 (0.01)  Ref | (1.13 - 3.03) | 1.89 (0.04)  Ref | (1.04 – 3.46) |
| Age | 14 – 25  25 – 45  > 45 | Ref  1.14 (0.60)  0.47 (0.02) | (0.70 - 1.84)  (0.26 - 0.87) | Ref  1.04 (0.89)  0.44 (0.04) | (0.56 – 1.94)  (0.20 – 0.98) |
| Education | Illiterate  0 – 5 years  5 – 10 years  > 10 years | 1.18 (0.64)  1.46 (0.22)  1.05 (0.86)  Ref | (0.59 - 2.38)  (0.80 - 2.69)  (0.63 - 1.74) | 0.83 (0.68)  1.31 (0.48)  0.81 (0.49)  Ref | (0.33 – 2.06)  (0.62 – 2.76)  (0.45 – 1.47) |
| BMI (kg/m^2^) | 18.5 – 25  < 18.5  > 25 | Ref  1.96 (0.003)  0.63 (0.45) | (1.25 - 3.08)  (0.19 - 2.09) | Ref  1.43 (0.19)  0.64 (0.55) | (0.84 – 2.42)  (0.15 – 2.75) |
| Smoking | Never  Ever | Ref  1.08 (0.76) | (0.68 - 1.71) |  |  |
| Alcohol Use | None  Any | Ref  1.49 (0.10) | (0.94 - 2.15) |  |  |
| Diabetes | Absent  Present | Ref  0.69 (0.09) | (0.45 - 1.06) |  |  |
| Baseline Smear | Negative Positive | Ref  1.70 (0.07) | (0.97 - 3.00) |  |  |
| Baseline Culture | Negative  Positive | Ref  2.18 (0.09) | (0.89 - 5.38) |  |  |
| Baseline Cavity | Present  Absent | Ref  1.04 (0.88) | (0.63 - 1.71) |  |  |
| Baseline X-ray Score (per 10 points) |  | 1.00 (0.33) | (0.99 - 1.10) |  |  |
| Month 2 Smear | Negative  Positive | Ref  2.13 (0.008) | (1.22 - 3.71) | Ref  1.73 (0.10) | (0.91 - 3.31) |
| Month 2 Culture | Negative  Positive | Ref  2.50 (0.0008) | (1.47 - 4.30) | Ref  2.06 (0.01) | (1.17 – 3.64) |
| Month 6 Cavity | Present  Absent | 2.59 (0.01)  Ref | (1.23 - 5.44) |  |  |
| Month 6 X-ray Score (per 10 points) |  | 1.02 (0.00024) | (1.01 - 1.03) |  |  |
| Antituberculosis Treatment Regimen | Daily  Intermittent | Ref  1.07 (0.84) | (0.57 - 2.00) |  |  |
| Baseline Smear Grade | 1+  2+  3+  Negative  Scanty | Ref  1.37 (0.34)  1.95 (0.07)  0.88 (0.72)  1.96 (0.11) | (0.71 – 2.65)  (0.94 – 4.06)  (0.45 – 1.75)  (0.86 – 4.45) |  |  |
| Month 2 Smear Grade | 1+  2+  3+  Negative  Scanty | Ref  -  -  0.95 (0.94)  2.86 (0.23) | -  -  (0.34 – 2.64)  (0.52 – 15.72) |  |  |

Abbreviations: HR, hazard ratio; BMI, body mass index

**Supplementary Table 3.** Comparison of characteristics of males who experienced recurrence compared to males who did not.

| Characteristic | Categories | Male with Recurrence  N = 75 (10%) | Male without Recurrence  N = 706 (90%) | P-value |
| --- | --- | --- | --- | --- |
| Baseline Characteristics |  |  |  |  |
| Study City | Chennai  Pune | 24 (32%)  51 (68%) | 347 (49%)  359 (51%) | 0.005 |
| Sex | Male  Female | - |  |  |
| Age (Years) | 14 – 25  25 – 45  > 45 | 15 (20%)  44 (59%)  16 (21%) | 113 (16%)  305 (43%)  288 (41%) | 0.003 |
| Education (Years) | Illiterate  0 – 5  5 – 10  > 10 | 10 (13%)  15 (20%)  32 (43%)  18 (24%) | 63 (9%)  106 (15%)  347 (49%)  189 (27%) | 0.32 |
| Body Mass Index  (kg/m^2^) | 18.5 – 25  < 18.5  > 25 | 21 (28%)  51 (68%)  3 (4%) | 309 (44%)  350 (50%)  46 (6%) | 0.01 |
| Smoking History | Never  Ever | 44 (59%)  25 (33%) | 398 (56%)  264 (37%) | 0.61 |
| Alcohol Use | None  Any | 26 (35%)  44 (59%) | 293 (42%)  393 (56%) | 0.45 |
| Diabetes | No  Yes | 52 (69%)  23 (31%) | 388 (55%)  318 (45%) | 0.02 |
| HIV | Negative  Positive | 55 (73%)  2 (3%) | 476 (67%)  13 (2%) | 0.67 |
| Baseline Smear | Negative Positive | 10 (13%)  65 (87%) | 148 (21%)  558 (79%) | 0.13 |
| Baseline Culture | Negative  Positive | 3 (4%)  72 (96%) | 71 (10%)  633 (90%) | 0.10 |
| Baseline Chest X-ray Cavity | Absent  Present | 37 (49%)  16 (21%) | 347 (49%)  196 (28%) | 0.45 |
| Baseline Smear Grade | 1+  2+  3+  Negative  Scanty | 15 (21%)  14 (19%)  8 (11%)  13 (17%)  7 (9%) | 122 (17%)  114 (16%)  64 (9%)  147 (21%)  42 (6%) | 0.73 |
| Antituberculosis Treatment Regimen | Daily  Thrice Weekly | 7 (9%)  66 (88%) | 101 (14%)  601 (85%) | 0.37 |
| Month 2 Smear | Negative  Positive | 47 (63%)  11 (15%) | 563 (80%)  64 (9%) | 0.05 |
| Month 2 Culture | Negative  Positive | 38 (51%)  16 (21%) | 531 (75%)  68 (10%) | 0.001 |
| Month 2 Smear Grade | 1+  2+  3+  Negative  Scanty | 2 (3%)  0 (0%)  -  44 (59%)  0 (0%) | 29 (4%)  2 (0%)  -  409 (58%)  5 (1%) | 0.88 |
| Month 6 Chest X-ray Cavity | Absent  Present | 20 (27%)  8 (11%) | 272 (39%)  32 (5%) | 0.01 |

REFERENCES

1.	Mave V, Meshram S, Lokhande R, et al. Prevalence of dysglycemia and clinical presentation of pulmonary tuberculosis in Western India. Int J Tuberc Lung Dis **2017**; 21(12): 1280-7.

2.	Gupte A, Padmapriyadarsini C, Mave V, et al. Cohort for Tuberculosis Research by the Indo-US Medical Partnership (CTRIUMPH): protocol for a multicentric prospective observational study. BMJ Open **2016**; 6(2): e010542.

3.	Kornfeld H, West K, Kane K, et al. High Prevalence and Heterogeneity of Diabetes in Patients With TB in South India: A Report from the Effects of Diabetes on Tuberculosis Severity (EDOTS) Study. Chest **2016**; 149(6): 1501-8.
